# HRCT Patterns of Drug-Induced Interstitial Lung Diseases: A Review

**DOI:** 10.3390/diagnostics10040244

**Published:** 2020-04-22

**Authors:** Giulio Distefano, Luigi Fanzone, Monica Palermo, Francesco Tiralongo, Salvatore Cosentino, Corrado Inì, Federica Galioto, Ada Vancheri, Sebastiano E. Torrisi, Letizia A. Mauro, Pietro V. Foti, Carlo Vancheri, Stefano Palmucci, Antonio Basile

**Affiliations:** 1 Radiology Unit 1, Department of Medical Surgical Sciences and Advanced Technologies “GF Ingrassia”-University Hospital “Policlinico-Vittorio Emanuele”, University of Catania, 95123 Catania, Italy; luigi.fanzone2@gmail.com (L.F.); monica.palermo91@gmail.com (M.P.); tiralongofrancesco91@hotmail.it (F.T.); salvatorecosentino209@gmail.com (S.C.); corry-16@hotmail.it (C.I.); federicagalioto91@gmail.com (F.G.); mauroletizia@tiscali.it (L.A.M.); pietrofoti@hotmail.com (P.V.F.); vancheri@unict.it (C.V.); spalmucci@sirm.org (S.P.); basile.antonello73@gmail.com (A.B.); 2 Department of Clinical and Experimental Medicine, University of Catania, Regional Referral Centre for Rare Lung Disease, 95123 Catania, Italy; adact1@hotmail.it (A.V.); torrisiseby@hotmail.it (S.E.T.)

**Keywords:** lung diseases, interstitial, multidetector computed tomography, idiopathic pulmonary fibrosis, toxicity, respiratory distress syndrome, acute

## Abstract

Interstitial Lung Diseases (ILDs) represent a heterogeneous group of pathologies, which may be related to different causes. A low percentage of these lung diseases may be secondary to the administration of drugs or substances. Through the PubMed database, an extensive search was performed in the fields of drug toxicity and interstitial lung disease. We have evaluated the different classes of drugs associated with pulmonary toxicity. Several different high resolution computed tomography (HRCT) patterns related to pulmonary drug toxicity have been reported in literature, and the most frequent ILDs patterns reported include Nonspecific Interstitial Pneumonia (NSIP), Usual Interstitial Pneumonia (UIP), Hypersensitivity Pneumonitis (HP), Organizing Pneumonia (OP), Acute Respiratory Distress Syndrome (ARDS), and Diffuse Alveolar Damage (DAD). Finally, from the electronic database of our Institute we have selected and commented on some cases of drug-induced lung diseases related to the administration of common drugs. As the imaging patterns are rarely specific, an accurate evaluation of the clinical history is required and a multidisciplinary approach—involving pneumologists, cardiologists, radiologists, pathologists, and rheumatologists—is recommended.

## 1. Introduction

Interstitial Lung Diseases (ILDs) represent a heterogeneous group of pathologies, characterized by high morbidity and mortality; they have been classified into four categories: (1) diseases with known causes, (2) Idiopathic Interstitial Pneumonias (IIPs), (3) granulomatous diseases (e.g., sarcoidosis and chronic hypersensitivity pneumonias), and (4) other or miscellaneous disorders. Drug-Induced Interstitial Lung Diseases (DILDs) have been included in the latter category, due to the fact that different radiological and morphological patterns have been associated to the administration of drugs [1,2]. 

Drugs may represent a possible etiological agent of damage, and the number of involved active substances has increased in recent years. As reported by Edwards and Aronson [1], an “Adverse Drug Reaction” (ADR) has been defined as “an appreciably harmful or unpleasant reaction, resulting from an intervention related to the use of a medicinal product, which predicts hazard from future administration and warrants prevention or specific treatment, or alteration of the dosage regimen, or withdrawal of the product” [3], and represents a common event in outpatients and hospitalized patients. In another study, ADR was considered responsible for ~6.5% of hospital admissions [4]. Although the most common manifestations involve gastrointestinal or metabolic system, pulmonary toxicity seems to be relatively uncommon [5], and it constitutes, cumulatively, less than 10% of the causes of hospitalization for ADR [6].

Many drugs and substances have been related to the possible onset of DILDs. It has been reported that DILDs constitute between 1.8% and 2.1% of the total number of ILDs in Italy, 2.6% in Germany and between 1.9%, and 3.5% of total ILDs in the USA [7]. Regardless, there are no definitive data and the real incidence of DILDs is probably still underestimated (Table 1 and Table 2).

The correct radiological approach to these disorders may represent an important element in the diagnostic path; an integrated and multidisciplinary approach is strongly recommended, in order to obtain accurate information on drug assumption (type, dose, and duration) from the clinical history of patients. However, pathogenesis, as well as real frequency, remains largely unknown [15].

The purpose of this paper is to illustrate the classes of drugs and the substances most frequently responsible for pulmonary toxicity; in addition, we provide a pictorial review of the most important radiological patterns, in order to provide a diagnostic address for radiologists.

## 2. Methods

Through the PubMed database, an extensive search was performed in the fields of drug toxicity and interstitial lung disease. We used the following keywords; “drug toxicity”, “Interstitial Lung Diseases”, “pulmonary toxicity”, “lung toxicity”, “adverse event”; no interval in the search period was specified. Our keyword research was conducted in January 2020. In our research, we have included only articles in English for which it has been possible to access the entire content; we excluded recurrent articles from the same authors and articles written in other languages. Relevant information was drawn from original articles, reference guidelines, and previous reviews; further work has been evaluated by analyzing the title, the abstract, and the bibliography of the articles found. Finally, publications relevant to the purpose have been selected for this review.

## 3. Results and Discussion

### 3.1. Etiopathogenesis and Risk Factors

Some of the possible risk factors for the development of pulmonary toxicity-related to drug exposure have been described in literature [2,15].

Although the pathology may have an idiosyncratic etiology, it is believed that the development of DILDs could be predicted by exposure to multiple toxic agents, chemotherapy, chronic inflammatory systemic diseases, chronic intestinal disorders, and old age [15].

In drugs with renal excretion, renal dysfunction is a risk factor for DILDs; for example, in the case of bleomycin, the risk of developing pulmonary toxicity, with fatal outcome, is significantly higher in patients with moderate chronic kidney disease than in patients with normal renal function, as shown in a non-recent retrospective analysis [16].

The impact of racial characteristics in severe ADR development is not well known. Recently, it has been shown that the cytochrome P450 polymorphism in the European population is significantly associated with an increased risk to develop DILDs [17]. As far as we know, the impact of ethnic factors on the development of DILDs has been validated only for some drugs, e.g., non-steroidal anti-inflammatory drugs (NSAIDs) pulmonary toxicity correlates with the expression of a specific HLA-DPB1 polymorphism gene in the Korean population [18].

Oxidative stress could be considered a definite risk factor for the onset and progression of tissue damage, and the administration of oxygen may increase active free radical exposure. In extreme cases, the consequences may be widespread acute alveolar damage, leading to the development of Acute Respiratory Distress Syndrome (ARDS). This risk factor has been described in particular with amiodarone and bleomycin [19].

Ionizing radiation is involved in the development of DILDs. It is known that radiation can be a cause of both acute or subacute damage and interstitial fibrosis; this toxic effect is considered an unavoidable consequence of chest radiation therapy [20]. Some chemotherapeutic agents, along with the use of oxygen (a known cause of interstitial damage), can exacerbate this condition, with consequent amplification of the damage.

The possible role of cumulative radiation dose in the induction of DILDs has been extensively investigated for amiodarone and, in smaller studies, for bleomycin and paclitaxel in combination with cyclophosphamide and cisplatin: for these drugs, the risk of interstitial disease correlates with the amount of radiation [21,22]. For other drugs, there seems to be no specific study in literature.

History of pre-existing pulmonary interstitial pathology probably represents an important risk factor for the development of DILDs, but a strong correlation has been described only for few drugs. In patients undergoing therapy with Gefitinib, previous ILDs represent a risk factor for the development of acute or chronic lung disorders and it leads to a worse prognosis, especially for older and smokers’ patients [23]. According to Sati et al. [24], in patients in continuous treatment with methotrexate, the incidence of DILDs is not increased, compared to the general population [24]. Leflunomide is correlated with an increased incidence of ILDs in patients having a simultaneous exposure to methotrexate and history of previous ILD, while there are no known data on other subgroups of patients [25].

The first case of heroin pulmonary toxicity was described by Osler in 1880; in years, a huge number of substances and drugs associated with possible DILD was observed and more than 350 different drugs are associated with iatrogenic lung diseases [26].

Some drugs, or their active metabolites, reach high concentrations in the lungs, and their accumulation is the cause of adverse effects. Another pathogenetic mechanism, not dose-related, is the development of a hypersensitivity reaction against the drug; the deposition of immunocomplexes can also involve the lung parenchyma, causing inflammatory reaction, and pulmonary and interstitial disease. According to the literature, drugs can also induce the production of intracellular toxicants, with consequent chronic cellular damage; this event has been described with regard to the induction of cell lipidosis by amiodarone [11]. Some authors have divided the etiology of DILDs in relation to the class of drugs involved; on this basis, we can summarily divide these classes into antibiotics, anti-inflammatory, biological, cardiovascular, antineoplastic, and various [11].

### 3.2. Antibiotics

Among antibiotics, it has been reported that those most frequently associated with lung toxicity are nitrofurantoin, sulfonamides, and sulfasalazine. Nitrofurantoin is a first-line drug in the treatment of urinary tract infections, particularly in females. Acute, subacute, and chronic forms of pulmonary toxicity have been described. The acute form has a relatively rare incidence, estimated at 1:5000 cases after the first exposure; however, due to its frequent use, acute toxicity is not a rare event overall. Renal dysfunction and old age are risk factors. Acute toxicity is, in almost all cases, represented by a hypersensitivity reaction that begins, regardless of dose, within one day after the first administration and tends to resolve spontaneously after discontinuation of the drug.

Chronic toxicity, which is associated with continued use of the drug and does not appear to be correlated with previous acute events, is more frequently represented by irreversible pulmonary fibrosis; less frequently, Organizing Pneumonia (OP) patterns can also occur [26,27]. Sulfalazina, which is used in inflammatory bowel disease and rheumatoid arthritis, is rarely associated with obliterating bronchiolitis and fibrosing alveolitis, and fatal cases have been reported [28]. Amino salicylic acid, which belongs to the class of sulfonamides, is rarely associated with pulmonary adverse reaction (<5% of patients), and the most common manifestations include alveolar infiltrates, lymphadenopathy, and pleural effusion; patients often suffer from angioedema, cough, and laryngeal edema [27].

### 3.3. Anti-Inflammatory and Immunomodulators Drugs

Various anti-inflammatory drugs have been associated with the development of DILDs. Penicillamine, gold compounds, methotrexate, and non-steroidal anti-inflammatory drugs (NSAIDs) have been correlated with the development of hypersensitivity pneumonitis, whereas colchicine and salicylates have been rarely reported as possible cause of ARDS.

Penicillamine has been found to be responsible for vasculitis, with pulmonary and renal involvement [29].

Acetyl-salicylic acid (Aspirin) can cause bronchospasm in 4% of the general population, and up to 25% in asthmatic patients [11]. The pathogenetic mechanism is probably related to the inhibition of cyclooxygenase and with dysregulated production of leukotrienes in predisposed subjects. Aspirin is also associated with the development of pulmonary edema [11]. A particular aspirin-induced toxic respiratory reaction is asthma.

Methotrexate is widely used as an immunomodulator in the treatment of rheumatoid arthritis and, less frequently, in other immunological disorders and proliferative diseases. Methotrexate pulmonary toxicity affects ~7% of patients undergoing chronic therapy, although rare cases of acute toxicity can occur in case of high doses administration [11]. A pre-existence of pulmonary pathology and the presence of renal failure have been considered risk factors for the development of pulmonary toxicity.

Pulmonary toxicity is generally characterized by a subacute onset and is characterized by being a Drug-Induced HyperSensitivity reaction (DIHS) in most cases [30,31].

**Figure 1:**
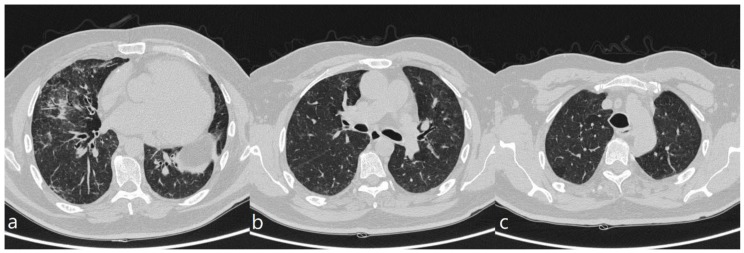
A patient with methotrexate induced lung toxicity. Axial scan passing through the bases (**a**), through the origin of the pulmonary artery (**b**), and through the apices (**c**). At the level of the lower lobes there are multiple areas of ground glass opacity. In this case, interlobular septa thickening and initial signs of lung architectural distortion are also evident: these findings are not commonly encountered in patients with methotrexate toxicity, but they have been also reported in literature. Therefore, they may represent a possible trap in the diagnosis.

Hypersensitivity pneumonitis is the most frequent type of pulmonary toxicity related to methotrexate; it is characterized by an interstitial lymphocyte infiltration with hyperplasia of the epithelial cells, small granulomatous formations, and, sometimes, areas of eosinophilic infiltration. Other methotrexate toxicity patterns include OP, Acute Interstitial Pneumonia, Pulmonary Fibrosis, and pleural effusion (Figure 1) [14].

Monoclonal antibodies are the main representatives of the class of biological drugs; toxicity has been reported most frequently for alemtuzumab, bevacizumab, cetuximab, rituximab, and trastuzumab [32]. Pulmonary toxicity related to these drugs, however, is rarely encountered.

To the best of our knowledge, few case reports have been published for lung disease related to rituximab, which is used in the treatment of some glomerulonephritis, lymphoproliferative diseases, and severe rheumatoid arthritis. Chest findings include diffuse bilateral lung infiltrates, Ground-Glass Opacity (GGO), alveolar hemorrhage, and alveolitis; toxicity mainly occurs in subacute form, about thirty days after the first drug administration [33]. The histological diagnoses, in those cases that underwent biopsy, include Organizing Pneumonia (OP), interstitial pneumonitis, HP, Idiopathic Pulmonary Fibrosis (IPF), alveolar-interstitial pneumonitis, diffuse alveolar damage, and desquamative interstitial pneumonia [34].

DILDs secondary to bevacizumab seem to be very rare. Bevacizumab is a monoclonal antibody that inhibits the activity of the vascular endothelial growth factor receptor, and it is indicated in the treatment of metastatic breast cancer, metastatic rectal carcinoma, and advanced lung cancer. Recently, the first case of interstitial disease has been described during maintenance therapy with bevacizumab in a patient with lung carcinoma [35]. Trastuzumab is a monoclonal antibody that binds the HER-2 receptor and it is prescribed in the treatment of advanced and metastatic breast carcinoma. This drug is considered to be relatively well tolerated and the indication of adverse events does not seem to increase significantly in association with other anti-neoplastic drugs. The most frequent respiratory adverse event appears to be bronchospasm. ILD is very rare, and only a single case with fatal outcome has been reported [36,37].

**Figure 2:**
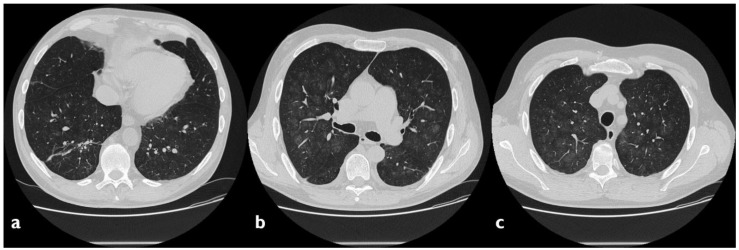
A case of suspected tocilizumab-induced lung toxicity. Axial scan passing through the bases (**a**), through the origin of the pulmonary artery, (**b**) and through the apices (**c**). Multiple areas of Ground-Glass Opacity (GGO), partly tending to confluence, predominantly located in the central regions of the lungs, partial sparing subpleural areas; fibrotic and nonspecific linear opacities are also shown in right lower lobe.

Tocilizumab is a monoclonal antibody that inhibits IL-6 receptors (sIL-6R and mIL-6R). IL-6 is a proinflammatory cytokine produced by a variety of cell types including T and B cells, monocytes, and fibroblasts, involved in various physiological processes, such as T cell activation and induction of immunoglobulin secretion. It is used, alone or in association with methotrexate, in the treatment of severe, active, and progressive rheumatoid arthritis, as well as in other autoimmune diseases. Pulmonary adverse reactions to tocilizumab include both infectious manifestations and non-infectious manifestation; among the latter, DILDs have been found, e.g., allergic pneumonitis, one case of IPF, and exacerbation of Rheumatoid Arthritis-Associated Interstitial Lung Disease (RA-ILD), as shown by Hadjinicolaou AV et al. [38]. In our experience, we found a case of tocilizumab toxicity, characterized—on HRCT images—by the presence of multiple areas of GGO, partly tending to confluence, showing partial sparing of subpleural areas (Figure 2).

### 3.4. Anti-Neoplastic Drugs

The prevalence of DILDs in patients receiving antiblastic therapies has been reported between 8% and 10%. Bleomycin is a widely used drug, alone or in combination with other treatments, of numerous neoplasms, including some lymphomas, germ cell tumors, and carcinomas. The incidence of DILDs during therapy with bleomycin is very high, up to 10% of cases, significantly higher than other antineoplastic drugs [12]. The development of an acute inflammatory process affecting the alveoli, with secondary activation of the fibroblasts, is the fundamental feature involved in the pathogenesis of lung injury; if exposure to bleomycin is chronic, deposition of collagen and hyaluronic acid is observed, while if the therapy is not prolonged over time, the damage is partially reversible. The risk of lung damage from bleomycin is dose-related, and exposure above 400 IU/m^2^ is associated with a 16% pulmonary disease rate in exposed patients [19]. The damage is usually subacute and the onset of respiratory symptom is usually four weeks after the star of therapy.

The most frequently described HRCT patterns are represented by Nonspecific Interstitial Pneumonia (NSIP), Usual Interstitial Pneumonia (UIP), and OP.

Alkylating agents represent a homogeneous group of drugs used in the treatment of numerous oncological diseases and autoimmune diseases. Busulfan is frequently associated with interstitial fibrosis, with a 4% incidence in chronic treatments and a relative risk that increases in relation to the duration of therapy.

**Figure 3:**
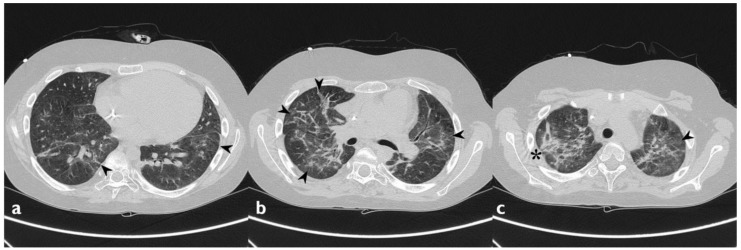
Cyclophosphamide-induced toxicity. Axial scan passing through the bases (**a**), through the origin of the pulmonary artery (**b**), and through the apices (**c**). Parenchymal consolidations are clearly recognizable in the upper lobes (black arrowheads); it is also possible to appreciate shaded areas of increased attenuation of the lung parenchyma as GGO spread to all segments (asterisk). Lung bases are less involved, as clearly depicted in figure a.

Cyclophosphamide is associated with DILDs in ~1% of cases when used as a single agent. Toxicity can have an early onset (1–6 months after the start of therapy), associated with reticulations, nodules, and areas of GGO in subpleural regions at the upper lobes (Figure 3) [39], or a late onset (after months or years of a low dose therapy), characterized by fibrotic reticulation, nodular opacities and loss of lung volume. Lower lobes are usually spared [40].

Pulmonary toxicity has also been described for chlorambucil and melphalan, but it is considered extremely rare [11].

Mitomycin is an antibiotic produced by Streptomyces Caespitosus, and it is used as a cytotoxic agent for the treatment of several solid neoplasia (including bladder, gastrointestinal tract, and breast cancer) and hematological malignancies. Pulmonary toxicity incidence varies, according to the authors, from 2% to 38%, and it is correlated with the cumulative dose and with radiotherapy [13]; mitomycin has been associated with different patterns of lung disease such as interstitial pneumonia, bronchiolitis, and pulmonary edema [19].

### 3.5. Cardiovascular Drugs

Among cardiovascular agents, amiodarone—widely used for the treatment of arrhythmias—is the drug that is most frequently associated with changes in respiratory function; an incidence ranging between 4% and 6% has been reported [41,42], with a mortality rate up to 20% [11]. Three different pathogenetic mechanisms have been reported: The first one is related to the nature of amphiphilic cationic that causes dysregulation of the activity of lysosomal enzymes, the second one to the activation of an aberrant immune response in a framework of lipoid pneumonia, and the third one to the induction of angiotensinogen expression.

Development of pulmonary toxicity may be multifactorial and associated with high concentrations of amiodarone and its active metabolite, desamethylamiodarone, in the lung parenchyma [42]. However, pulmonary toxicity appears to be related to both dose and exposure [41]. Symptomatology is variable and nonspecific, and it can be represented by non-productive cough, dyspnea, ARDS, and in severe cases, death [11].

**Figure 4:**
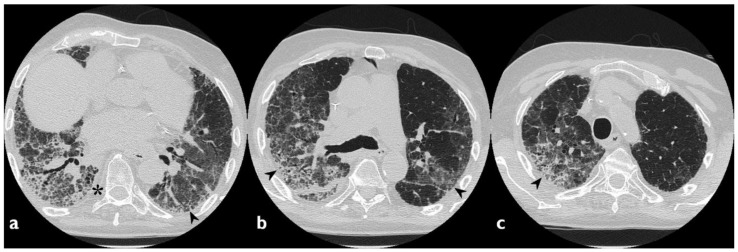
A case of Amiodarone-Induced Lung Toxicity (AILT). Axial scan passing through the bases (**a**), through the origin of the pulmonary artery (**b**), and through the apices (**c**). Reticulations, traction bronchiectasis, and widespread areas of GGO are shown in panels a–c (black arrowheads); parenchymal alterations have central and peripheral distribution. At the bases, in the subpleural field, an initial honeycomb pattern is appreciable (asterisk).

**Figure 5:**
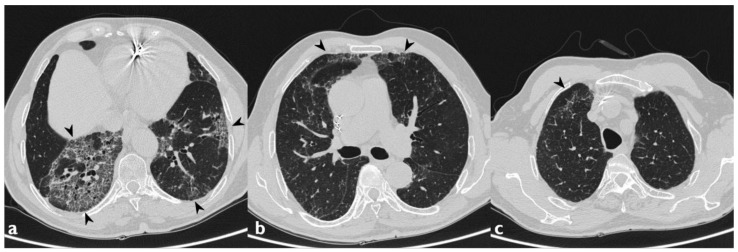
Another case of AILT. Axial scan passing through the bases (**a**), through the origin of the pulmonary artery (**b**), and through the apices (**c**). Reticular interstitial pattern superimposed to areas of GGO, distributed mainly to the lower lobes bilaterally and at lingula (black arrowheads); multiple traction bronchiectasis and bronchioloectasie are also present.

**Figure 6:**
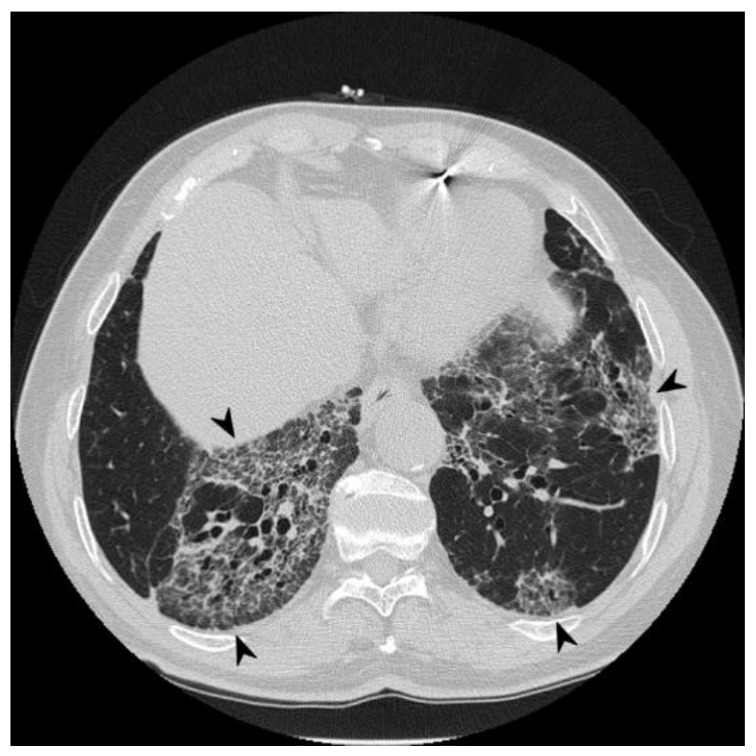
Same patient as the previous figure, follow-up two years after discontinuation of therapy: the scans passing through the basal segments demonstrate the substantial stability of the radiological picture (black arrowheads indicate the previous findings).

**Figure 7:**
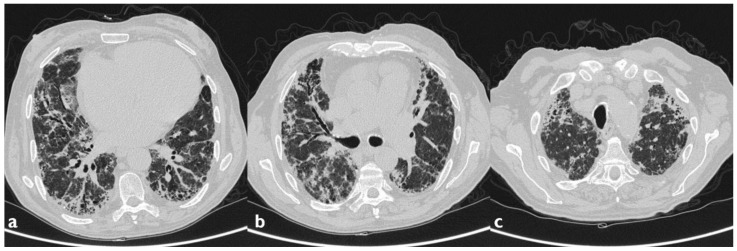
Another case of AILT. Axial scan passing through the bases (**a**), through the origin of the pulmonary artery (**b**), and through the apices (**c**). Diffuse reticular interstitial pattern and GGO, with multiple bronchiectasis and subpleural consolidation areas; these morphological features resemble a Nonspecific Interstitial Pneumonia (NSIP) pattern secondary to drug toxicity.

**Figure 8:**
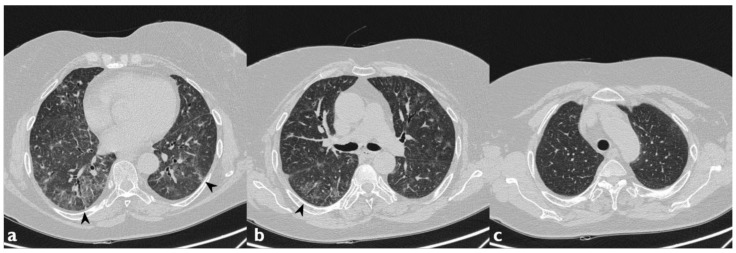
Another case of AILT. Axial scan passing through the bases (**a**), through the origin of the pulmonary artery (**b**), and through the apices (**c**). Interstitial disease with NSIP pattern. Diffuse increase in density of the lung parenchyma with a GGO appearance (black arrowheads indicate the previous findings).

The HRCT characteristics of full-blown forms are bilateral infiltrates with high alveolar and interstitial attenuation; in the initial stages, GGO in the peripheral areas can be found [43,44]. Pleural thickening, basal reticulation, and traction bronchiectasis are typical of the forms with a fibrous course (Figure 4, Figure 5, Figure 6, Figure 7 and Figure 8). The high attenuation of lung infiltrates has been linked to the presence of iodine in the drug [44].

Lung toxicity has also been reported for tocainide and flecainide, two other widely used antiarrhythmic agents. In particular, very rare cases of ARDS have been reported for flecainide [45], while cases of acute interstitial pneumonitis (IP) and pulmonary fibrosis [46] have been reported for tocainide.

Inhibitors of angiotensin-converting enzyme (ACE-I) are widely used as antihypertensive drugs and in the treatment of some nephropathies; it is known that these drugs are frequently responsible for dry and continuous cough (up to 35%) [47]. However, there are no known interstitial disorders certainly associated with this class of drugs. Recent evidence, inter alia, suggests a protective role from radiation-induced lung fibrosis [20].

### 3.6. Psychoactive Drug

Sporadic cases of ILDS are associated with these substances, especially with cocaine and more rarely opioids.

**Figure 9:**
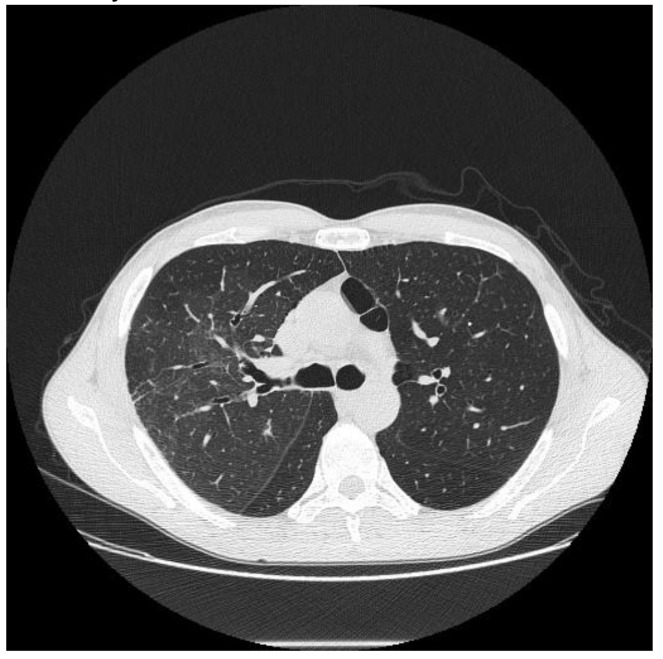
Lung cocaine toxicity in a patient admitted to the emergency department with hemoptysis and dyspnea 24 h after inhalation of cocaine. Focal area of GGO, smooth septal thickening, and centrilobular nodule are visible in the right upper lobe.

**Figure 10:**
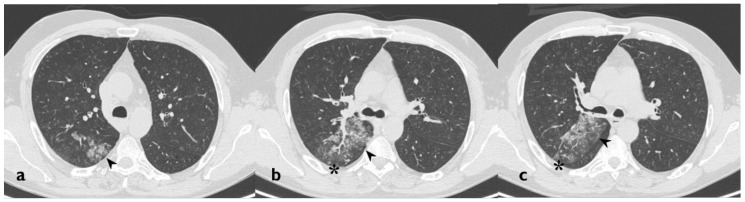
Another case of lung cocaine toxicity. Axial scan passing through the upper lobes (**a**–**c**). Focal area of GGO (black arrowheads), centrilobular nodule, and the tree-in-bud pattern (asterisk) are visible in the right upper lobe.

Cocaine is an alkaloid with anesthetic properties obtained from the leaves of *Erythroxylon coca*. It is a widely abused substance, because of its strong sympathomimetic and central nervous system stimulant effects, which are due to its capacity to interfere with the reuptake of catecholamines and serotonin. HRCT patterns associated with cocaine toxicity include GGO (reported in 100% of the cases) (Figure 9); consolidations (50%); the halo sign (25%); as well as smooth septal thickening, paraseptal emphysema, centrilobular nodules, and the tree-in-bud pattern (Figure 10) [48]; however, as many cocaine abusers are also marijuana or tobacco smokers, it is difficult to determine which alterations are specifically caused by cocaine [49].

Alterations provoked by cocaine can be divided in acute or chronic. “Crack lung” refers to a hemorrhagic alveolitis and signs of diffuse alveolar damage that occurs within 48 h of smoking crack; in these cases, GGO and radiological features of DAD may be appreciated on HRCT images. Biopsy or autopsy specimens show alveolar damage and hyaline membrane formation; eosinophilia can be associated [50]. Acute eosinophilic pneumonia is occasionally reported within hours to days after cocaine use [51].

Chronic use of cocaine is associated with a variety of histopathologic changes in the lung, including foreign body granulomatosis—e.g., talc, silica, cotton fibers, and others—bronchiectasis, and recurrent alveolar hemorrhage. Interstitial pneumonitis with accumulation of free silica within histiocytes has been reported in a crack cocaine smoker [52]. Recurrent alveolar hemorrhage from prolonged crack cocaine use has been associated with the development of severe pulmonary fibrosis [53].

### 3.7. Morphological Patterns and Imaging Evaluation

Various morphological HRCT patterns have been described in the literature. Among the described patterns, we can mention interstitial fibrosis with NSIP features or, more rarely, with UIP features, hypersensitive pneumonitis (HP), OP—the latter in the past reported with the term of “Obliterating Bronchiolitis Organizing Pneumonia” (BOOP in older works)—and ARDS.

**Figure 11:**
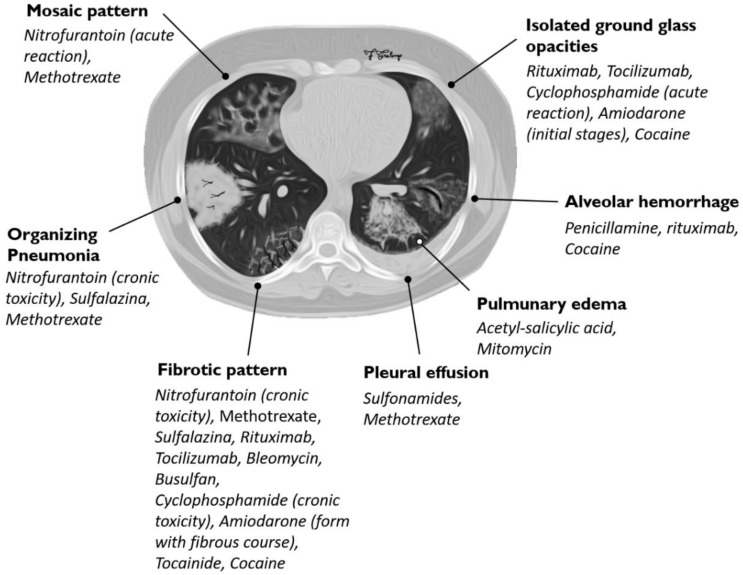
Association between HRCT patterns and the drugs most frequently responsible for lung toxicity.

HRCT pattern could be predictive of the histological one, especially in chemotherapeutic drugs [27]. It is important to note that imaging patterns are often nonspecific and, moreover, different drugs may be responsible for different forms of toxicity (Table 3, Figure 11). The heterogeneity of imaging patterns, which may be associated to the administration of a single drug, has been clearly reported in literature [15,31]. Clinicians and radiologists may often verify that a single drug could be associated to different imaging patterns of toxicity, as clearly evident in the “Pneumotox on line” website, which represents a very helpful guide in the evaluation of DILDs [54]. Methotrexate, for example, has been associated to the following different categories of pathological processes; (1) interstitial lung diseases, (2) acute lung injury, (3) alveolar hemorrhage, (4) airway involvement, (5) pleural or pericardial involvement, (6) vasculopathies, (7) mediastinal involvement (lymphadenopathy), and (8) central-large-upper airway involvement [54].

On the other hand, a clinical manifestation or a pathological process could be reproduced by several imaging patterns: a pulmonary injury-consisting of eosinophilic pneumonia may be represented by organizing pneumonia or HP pattern [15].

There are different mechanisms that could explain the high overlap and heterogeneity, such as epigenetic, genetic factors, ethnicity, dose, drug interactions, oxidative stress, exposure to ionizing radiations (for both diagnostic and therapeutic purposes), and renal dysfunction (due to impaired excretion of metabolites) [15,16,19,21,22]; the role of underlying lung disease must also be taken into account [15].

Drug-induced NSIP is one of the most frequent forms of ILD; it is characterized by the presence of GGO—mainly subpleural—in lower lobes, with patchy or diffuse thickening of intralobular septa; the lesions have a more homogeneous distribution than in other etiologies.

UIP is characterized by cystic airspace with thick wall, areas of honeycombing with a prevalent basal and subpleural distribution, and diffuse traction bronchiectasis [55].

OP is characterized by the presence of inflammatory infiltrates in the respiratory bronchioles and alveoli, patchy airspace consolidations with air bronchograms sign without signs of fibrosis, traction bronchiectasis, or honeycombing; lesions are commonly peripheral.

In HP there are GGO, mosaic patterns, and, in chronic forms, areas of honeycombing and fibrosis located in the upper lobes and characterized by peribronchovascular distribution [56]. In cases of hypersensitivity pneumonia, after excluding other causes, it is necessary to consider that the drug responsible for interstitial disease behaves as an antigen and causes an inappropriate immune response; this immune response is responsible of the bronchiolitis (with lesions of the terminal airways), which justifies the air trapping that HRCT imaging manifests as a mosaic pattern. The mosaic attenuation pattern reflects air trapping from underlying obliterative bronchiolitis

ARDS is related to lung injury of acute onset and is characterized by symmetrical pulmonary opacification with anteroposterior density gradient and GGO areas. Drug-induced hypersensitivity syndrome is a severe form of immune-mediated reaction with acute or subacute onset following drug assumption. It has a systemic involvement, and lung toxicity can manifest as Loffler’s syndrome or as an eosinophilic pneumonia. Loffler’s syndrome manifests clinically with cough, dyspnea, fever, and rash; radiological findings include pulmonary infiltrates and GGO with a bilateral distribution and predominantly in the upper lobes [14]. The presence of granulomatous lesions represents a rare form of toxicity described for immunomodulatory biological agents such as infliximab and etanercept. The pattern is characterized by the presence of sarcoidosis-like bilateral pulmonary infiltrates and non-necrotizing granulomas surrounded by variable signs of fibrosis; a similar pattern has also been described for sulfasalazine [57].

Hemorrhagic alveolitis is an uncommon manifestation of pulmonary drug toxicity. Histologically, it is represented by the presence of blood cells in the context of alveolar spaces, due to damage to the alveolar basement membrane subsequent to an allergic reaction. As reported by Schwarz et al., there are several pathological causes associated with drug-induced alveolar hemorrhage: (1) small vessel vasculitis—also called pulmonary angiitis—(diphenylhydantoin, propylthiouracil, and all trans-retinoic acid), (2) veno-occlusive pulmonary disease, (3) hypersensitivity reaction, and (4) direct pulmonary epithelial toxicity that produces diffuse alveolar damage [58]. Alveolar hemorrhages can also occur during treatment with anticoagulants. The symptomatology is characterized by cough, hemoptysis, dyspnea and anemia [14]. Drug-induced Lupus (DIL) is a further, not uncommon, form of systemic toxicity with possible pulmonary involvement. It can be associated with the chronic use of many commonly used drugs that induce the formation of auto-antibodies, even in non-symptomatic patients. Symptoms and laboratory test results are similar to primitive LES: joint pain, erythema, photosensitivity, neurological and renal dysfunction, fatigue, and constitutional symptoms. Pulmonary involvement is represented in most cases by pleural effusion and, more rarely, by bilateral pulmonary infiltrates and fibrosis [59].

### 3.8. Diagnosis

The diagnosis of DILDs is not simple and requires the integration of clinical, laboratory, and imaging data. Histological examination demonstrates substantially identical characteristics between idiopathic and drug-related forms; physical, laboratory, and radiological findings are not able, separately, to resolve the diagnostic questions. Laboratory analysis is necessary to rule out infectious causes and autoimmune or rheumatological diseases; however, these data should be interpreted with caution because some anti-infectives and immunomodulators can induce significant lung injury themselves. Infections can however be excluded by the execution of cultural examinations. Bronchoalveolar lavage (BAL) can be useful in the diagnostic process. In patients with hypersensitivity pneumonia, lymphocytosis >50% and a decreased CD4/CD8 ratio could be found. Eosinophilic pneumonia is characterized by an elevated BAL eosinophil count >25% [58]. Evaluation of respiratory function parameters, e.g., FEV1, FEV1/FVC ratio, and DLCO, can help to define a restrictive or obstructive pattern, but obviously the results are superimposable to idiopathic interstitial diseases. HRCT appears to be the most sensitive radiological examination to make a diagnosis of interstitial disease, but it should be performed and evaluated by radiologists with experience in pulmonary diseases and in a multidisciplinary approach, as suggested by the guidelines [9,60,61]. In this rather complex and uncertain context, the resolution of diagnostic doubt is represented by the possible cause-effect relationship between the onset of a pulmonary pathology and exposure to a drug; it is therefore useful to evaluate the recent pharmacological anamnesis and investigate any previous ones [14]. In acute manifestations, drug discontinuation sometimes improves the clinical outcome: this eventuality may suggest the correct diagnosis. HRCT can be useful in evaluating the follow up of cases of acute toxicity in relation to the possible regression of the signs previously encountered, after discontinuation of the drug; in patients with chronic interstitial disease, the role is more uncertain due to the irreversibility of fibrotic changes.

However, the diagnosis of drug toxicity remains a diagnosis by exclusion, and other concomitant interstitial diseases or infectious diseases must be taken into consideration.

### 3.9. Management

The therapy for DILDs is rather poor: the suspension of the injurious drug should be considered in favor of substitution with another drug. The suspect drug should be avoided in future treatments. In chronic forms, the fibrotic lesions are essentially irreversible, regardless of the suspension of the drug. In OP and HP, cortisone may play a role in the correction of symptoms and in accelerating the resolution of the clinical picture. The induction of hyposensitization can play a role in the case of non-substitutable drugs or when the alternatives are not well tolerated [27].

## 4. Conclusions

The diagnosis of DILDs are constantly increasing, also following the introduction of new drug therapies. The morphological HRCT patterns are not pathognomonic and therefore the diagnosis should be based on clinical-anamnestic data, radiological, and laboratory features. An early diagnosis of DILDs may be useful for the treatment of the disease. HRCT is therefore an indispensable tool for the diagnosis and for the improvement of patient outcomes.

## Figures and Tables

**Table 1:** Drugs most commonly responsible for Drug-Induced Interstitial Lung Diseases (DILDs) and estimated incidence.

Drugs	Estimated Incidences	References
Nitrofurantoina	1 on 5000 (acute toxicity)	[8]
Acetyl-salicylic acid	From 4% (general adult population) to 25% (asthmatic patients)	[9]
Amiodarone	6%	[10]
Methotrexate	7% (chronic toxicity), very rare (acute toxicity)	[11]
Bleomycin	10%	[12]
Busulfan	4%	[9]
Mitomycin	2–38%	[13]
Cyclofosphamide	1% (when used as single agent)	[9]

**Table 2:** Association between pathological appearance and drug administered.

Pattern	Associated Drugs	References
OP	Amphotericin-B, Amiodarone, Bleomycin, Doxorubicin, Interferon, Metotrexatem, Mitomycin, Nitrofurantonina, Phenytoin, Ticlopidine, Tryptophan, Sulphalazine	[14]
HP	Ampicillin, Bupropion, Carbamazepine, Ciprofloxacin, Citarabine, Cephalosporins, interferon-alpha, sulfonamides, ticlopidine, trimethoprim-sulfamethoxazole, sirolimus	[9]
Interstitial pneumonia	Adalimumab, Amphotericin B, Amiodarone, Azathioprine, Bleomycin, Busulfan, Chlorambucil, Cyclofosphamide, Etanercept, Flecainide, Interferon alfa, Interferon beta, Infliximab, Melphalan, Methadone, Metotrexate, Nitrofurantoin, Paclitaxel, Penicillamine, Rituximab, Sirolimus, Statine, Sulfasalazine	[14]
Loeffler syndorme	Amiodarone, ASA, Bleomycin, Carbamazepine, Captopril, Ibuprofen, Imipramine, Isoniazide, Metotrexate, GM-CSF, Naproxen, Gold salts, Sulfasalazine, Procarbazine, Penicillins, Tryptophans, Zafirleukast	[11]
Pulmonary edema	Amlodipine, ASA, Cyclosporine, Citarabine, Chlorothiazide, Clozapine, Heroin, Epinephrine, Gemcitabine, Ketoprofen, Interleukin, Methadone, Metotrexate, Mitomycin, Nitric Oxide, Propanolol, Verapamil	[14]
ARDS	Amiodarone, Citarabine, Immunoglobulins, GM-CSF, Nitrofurantoin, Infliximab, Talc, Vinblastine, Vincristine	[14]

**Table 3:** Association between HRCT patterns and the drugs most frequently responsible for lung toxicity.

HRCT Pattern	Associated Drugs
Fibrotic pattern	Nitrofurantoin (chronic toxicity), methotrexate, sulfalazina, rituximab, tocilizumab, bleomycin, busulfan, cyclophosphamide (chronic toxicity), amiodarone (form with fibrous course), tocainide, cocaine
Organizing pneumonia	Nitrofurantoin (chronic toxicity), methotrexate
Mosaic pattern	Nitrofurantoin (acute toxicity), methotrexate, sulfalazina
Isolated ground glass	Rituximab, tocilizumab, cyclophosphamide (acute reaction), amiodarone (initial stage), cocaine
Alveolar hemorrhage	Penicillamine, rituximab, cocaine
Pulmonary edema	Acetyl-salicylic acid, mitomycin
Pleural effusion	Sulfonamides, methotrexate
